# Supplementary material for: Early immune markers of clinical, virological, and immunological outcomes in patients with COVID-19: a multi-omics study
Source: eLife. 2022 Oct 14;11:e77943. doi: 10.7554/eLife.77943 (PMC9566856; doi:10.7554/eLife.77943)
Supplement: Supplementary file 1. [file elife-77943-supp1.docx]

Supplemental File 1: Characteristics of Study Participants (Lambda study)

|  | **Treatment arm** | |  |
| --- | --- | --- | --- |
|  | **Lambda (N=54)** | **Placebo (N=54)** | **Overall (N=108)** |
| **Age in years**, median (range) | 37 (18-65) | 36 (20-71) | 37 (18-71) |
| **Male**, n (%) | 32 (59.3%) | 30 (55.6%) | 62 (57.4%) |
| **Race / Ethnicity**, n (%) |  |  |  |
| Latinx | 30 (56.7%) | 37 (68.3%) | 67 (62.0%) |
| White | 18 (30.0%) | 12 (25.0%) | 30 (27.8%) |
| Asian | 4 (5.0%) | 5 (6.7%) | 9 (8.3%) |
| Native Hawaiian or other Pacific Islander | 2 (3.3%) | 0 (0%) | 2 (1.9%) |
| **BMI (kg/m^2^)**, median (IQR) | 27.6 (25.4-31.1) | 28.5 (24.8-32.3) | 27.7 (24.9-32.0) |
| **Comorbid conditions** |  |  |  |
| Hypertension | 9 (16.7%) | 5 (6.8%) | 14 (13.0%) |
| Diabetes | 4 (7.4%) | 8 (14.8%) | 12 (11.1%) |
| Asthma | 2 (3.7%) | 2 (3.7%) | 4 (3.7%) |
| Heart Disease | 3 (4.1%) | 1 (1.9%) | 4 (3.7%) |
| **Asymptomatic at baseline**, n (%) | 5 (9.3%) | 3 (5.6%) | 8 (7.4%) |
| **Duration of symptoms in days prior to randomization**, median (IQR) ^1^ | 4 (3-6) | 5 (3-5) | 5 (3-6) |
| **Baseline oropharyngeal SARS-CoV-2 cycle threshold, median (IQR) ^2^** | 30.9 (26.4-33.8) | 29.3 (26.4-34.3) | 30.3 (26.4-34.3) |
| **Baseline SARS-CoV-2 IgG seropositivity**, n (%) | 17 (31.5%) | 24 (44.4%) | 41 (38.0%) |
| **Outcomes after Randomization** |  |  |  |
| Days until viral shedding cessation, median  (IQR) | 7 (5-13) | 7 (5-10) | 7 (5-13) |
| Hospitalization by day 28, n (%) | 2 (3.7%) | 2 (3.7%) | 4 (3.7%) |
| Hospitalization and/or emergency room  visit by day 28, n (%) | 5 (9.3%) | 3 (5.6%) | 8 (7.4%) |
