# Supplementary material for: Early immune markers of clinical, virological, and immunological outcomes in patients with COVID-19: a multi-omics study
Source: eLife. 2022 Oct 14;11:e77943. doi: 10.7554/eLife.77943 (PMC9566856; doi:10.7554/eLife.77943)
Supplement: Supplementary file 4. [file elife-77943-supp4.docx]

##### **Supplemental File 4: ICS antibody panel**

| **ICS Antibody Panel** | | | | | |
| --- | --- | --- | --- | --- | --- |
| **Surface Antibodies** | **Fluorochrome** | **Clone** | **Vendor** | **Catalog** | **Amount Per 50 uL** |
| CCR7 | BV421 | G043H7 | BioLegend | 353208 | 2.5uL |
| CD14  CD19   LIVE/DEAD | BV510  BV510  Aqua | M5E2  HB19 | BioLegend  BioLegend  Invitrogen | 301842  302242  L34965 | 0.5uL  0.5 uL  0.25uL |
| CD45RA | BV605 | HI100 | BioLegend | 304134 | 0.4uL |
| CD4 | BV650 | RPA-T4 | BioLegend | 300536 | 1uL |
| CD8A | BV785 | RPA-T8 | BioLegend | 301046 | 1uL |
| CD107A | FITC | H4A3 | BioLegend | 328606 | 1uL |
| CD3 | APC-H7 | SK7 | BD | 560176 | 2.5uL |
|  |  |  |  |  |  |
| **Intracellular Antibodies** | **Fluorochrome** | **Clone** | **Vendor** | **Catalog** | **Amount Per 50 uL** |
| IFN-g | PerCP Cy5.5 | 4S.B3 | BioLegend | 502526 | 0.5uL |
| IL-21 | eFluor660 | eBio3A3-N2 | eBioscience | 50-7219-42 | 1.25uL |
| TNF | AF700 | MAb11 | BD | 557996 | 0.5uL |
