# Supplementary material for: Early immune markers of clinical, virological, and immunological outcomes in patients with COVID-19: a multi-omics study
Source: eLife. 2022 Oct 14;11:e77943. doi: 10.7554/eLife.77943 (PMC9566856; doi:10.7554/eLife.77943)
Supplement: Supplementary file 5. [file elife-77943-supp5.docx]

Supplemental Table 5: Characteristics of Validation Study Participants (Placebo arm, Favipiravir trial)

| **Characteristic** | **N=54** |
| --- | --- |
| **Age in years**, median (range) | 44 (20-73) |
| **Male**, n (%) | 26 (48.2%) |
| **Race / Ethnicity**, n (%) |  |
| Latinx | 20 (39.2%) |
| White | 22 (43.1%) |
| Asian | 5 (9.8%) |
| Native Hawaiian or other Pacific Islander | 1 (2.0%) |
| Other | 3 (5.9%) |
| **BMI (kg/m^2^)**, median (IQR) | 28.6 (24.8-32.7) |
| **Comorbid conditions** |  |
| Hypertension | 5 (15.0) |
| Diabetes | 3 (6.7) |
| Chronic lung disease | 3 (3.3) |
| **Asymptomatic at baseline**, n (%) | 1 (1.9%) |
| **Duration of symptoms in days prior to randomization**, median (IQR) ^1^ | 5 (4-7) |
| **Baseline oropharyngeal RT-PCR positivity, n (%)** | 50 (87·7) |
| **Clinical Outcomes after Randomization** |  |
| Hospitalizations by day 28, n (%) | 4 (7.4%) |
| Hospitalizations and/or emergency room visits by day 28, n (%) | 7 (13.0%) |
